# Incorporating machine learning into the decision-making process regarding implantable cardioverter-defibrillator treatment

**DOI:** 10.1093/europace/euae058

**Published:** 2024-02-28

**Authors:** Ezgi Çamlı Babayiğit, Erdi Babayiğit, Bülent Görenek

**Affiliations:** Cardiology Department, Eskişehir Şehir Hastanesi, 71 Evler, Çevre Yolu, 26080 Odunpazarı/Eskişehir, Eskişehir 26080, Turkey; Cardiology Department, Eskişehir Şehir Hastanesi, 71 Evler, Çevre Yolu, 26080 Odunpazarı/Eskişehir, Eskişehir 26080, Turkey; Cardiology Department, Eskişehir Şehir Hastanesi, 71 Evler, Çevre Yolu, 26080 Odunpazarı/Eskişehir, Eskişehir 26080, Turkey

## Dear editor,

We have read with interest the research paper entitled ‘Optimizing patient selection for primary prevention implantable cardioverter-defibrillator implantation: utilizing multimodal machine learning to assess risk of implantable cardioverter-defibrillator non-benefit’ by Kolk M.Z.H. et al.^1^ The aim of the study was to predict the risk of non-arrhythmic death in patients with implantable cardioverter-defibrillator (ICD) implantation for primary prevention through serial 12-lead electrocardiogram recordings and clinical variables. Here, we would like to draw your attention to a few points of the study.

In predicting the benefit from defibrillator treatment, data such as patients’ The New York Heart Association (NYHA) functional classes, left ventricular ejection fraction (LVEF) values, the presence of ventricular aneurysm or scar tissue detected by imaging methods, history of syncope, and atrial fibrillation (AF) have been shown to be useful.^2,3^ Providência et al.^4^ revealed that using NYHA, LVEF, and AF in risk scoring can be useful in identifying those who will benefit from defibrillator therapy. Adding these data, which have been shown to be useful in past studies, to multimodal machine learning could contribute to the study.

However, it is worth noting that the time span for gathering study data (between 2007 and 2021) is quite extensive, considering the rapid development of device technology during this period. Advances in transvenous systems, anti-tachycardia pacing, and programming configurations, have significantly altered the risk–benefit ratio for patients.^5^ Furthermore, the study lacks information regarding device programming and monitoring details, which could have significant implications on the study results.

The question of who will benefit from ICD treatment has led clinicians to develop various scores in recent years. This study has demonstrated that incorporating machine learning into the decision-making process regarding ICD treatment would be beneficial. We thank the researchers who contributed to solving this problem.

## Data Availability

The data underlying this article will be shared upon reasonable request to the corresponding author.
